# Mechanical and IL-1β Responsive miR-365 Contributes to Osteoarthritis Development by Targeting Histone Deacetylase 4

**DOI:** 10.3390/ijms17040436

**Published:** 2016-03-23

**Authors:** Xu Yang, Yingjie Guan, Shaoqi Tian, Yuanhe Wang, Kang Sun, Qian Chen

**Affiliations:** 1 Department of Orthopedics, the Affiliated Hospital of Qingdao University, 1677 Wutaishan Rd, Qingdao 266000, China; xu_yang0608@outlook.com (X.Y.); shaoqi99@aliyun.com (S.T.); wangyuanhe-2004@163.com (Y.W.); 2 Cell and Molecular Biology Laboratory, Department of Orthopedics, Alpert Medical School of Brown University/Rhode Island Hospital, Providence, RI 02903, USA; yingjie_guan@brown.edu (Y.G.); qian_chen@brown.edu (Q.C.)

**Keywords:** miR-365, osteoarthritis, mechanical loading, inflammation

## Abstract

Mechanical stress plays an important role in the initiation and progression of osteoarthritis. Studies show that excessive mechanical stress can directly damage the cartilage extracellular matrix and shift the balance in chondrocytes to favor catabolic activity over anabolism. However, the underlying mechanism remains unknown. MicroRNAs (miRNAs) are emerging as important regulators in osteoarthritis pathogenesis. We have found that mechanical loading up-regulated microRNA miR-365 in growth plate chondrocytes, which promotes chondrocyte differentiation. Here, we explored the role of the mechanical responsive microRNA miR-365 in pathogenesis of osteoarthritis (OA). We found that miR-365 was up-regulated by cyclic loading and IL-1β stimulation in articular chondrocytes through a mechanism that involved the transcription factor NF-κB. miR-365 expressed significant higher level in rat anterior cruciate ligament (ACL) surgery induced OA cartilage as well as human OA cartilage from primary OA patients and traumatic OA Patients. Overexpression of miR-365 in chondrocytes increases gene expression of matrix degrading enzyme matrix metallopeptidase 13 (MMP13) and collagen type X (Col X). The increase in miR-365 expression in OA cartilage and in response to IL-1 may contribute to the abnormal gene expression pattern characteristic of OA. Inhibition of miR-365 down-regulated IL-1β induced MMP13 and Col X gene expression. We further showed histone deacetylase 4 (HDAC4) is a direct target of miR-365, which mediates mechanical stress and inflammation in OA pathogenesis. Thus, miR-365 is a critical regulator of mechanical stress and pro-inflammatory responses, which contributes cartilage catabolism. Manipulation of the expression of miR-365 in articular chondrocytes by miR-365 inhibitor may be a potent therapeutic target for the prevention and treatment of osteoarthritis.

## 1. Introduction

Osteoarthritis (OA) is a degenerative joint disease characterized by degradation of articular cartilage, thickening of subchondral bone, and formation of osteophytes. It is among the most important causes of pain, disability, and economic loss in all populations [1]. Currently, there is no effective disease modifying treatment for OA until the end stage of disease necessitating joint replacement. The molecular pathogenic mechanisms of OA are still unclear and remain an active area of investigation as targets for preventive and disease modifying therapies are greatly needed. Biophysical and biochemical factors, including mechanical stress and pro-inflammatory cytokines, respectively, are responsible for disruption of this homeostasis and initiation of the catabolic pathway [2,3,4]. It is known that chondrocytes that undergo a hypertrophy-like phenotype produce OA related changes and play an important role in onset and development in OA [5]. However, the molecular pathogenic mechanisms leading to altered gene expression in OA need to be elucidated in more detail.

Recently, it was proposed that epigenetic mechanisms play a role in modulating cell phenotype in OA resulting in permanent changes in DNA transcription. One of the epigenetic mechanisms involved is based on microRNA (miRNA) expression [6,7]. miRNAs are short (19 to 23 nucleotides) non-coding RNAs, endogenously produced and function as posttranscriptional negative regulators by promoting messenger RNA (mRNA) degradation or repressing translation through complementarily binding target sequences in the 3′-untranslated regions (3′-UTRs) of specific mRNA targets [8,9]. Hundreds of miRNAs have been identified in various organisms, and many are evolutionarily conserved. Evidence has revealed that miRNAs are associated with cartilage development and diseases. Mice with global reduction in miRNAs by deleting Dicer in growth plate chondrocytes reduced limb size, causing a severe skeletal growth defect and early postnatal lethality. Deletion of microprocessor Complex Subunit Drosha or DiGeorge syndrome chromosomal (or critical) region 8 (DGCR8) in growth plate chondrocytes caused a lethal skeletal defect similar to that of Dicer deletion. Early postnatal Drosha deficiency induces articular chondrocyte death and can cause a mild OA-like pathology [10,11,12]. Furthermore, more and more individual miRNAs, including miR-27b, miR-34a, miR-140, and miR-146a, have been linked to arthritis pathogenesis [6,13,14]. miR-140, which was originally found in cartilage, has been linked more specifically to OA [15,16]. Of importance, miR-140 decreases the expression of genes known to play detrimental roles in OA cartilage. Targeted deletion of miR-140 in mice resulted in age-related OA-like changes [17]. miR-140 expression is significantly decreased in human OA chondrocytes [16], thus favoring an increased expression of its target genes and consequently a role in cartilage degradation. miR-146a has also received much attention since 2008 although its function in OA pathology is still controversial. Li *et al.* proposed that expression of miR-146a in chondrocytes contributes to OA pathogenesis by diminishing the response to transforming growth factor β (TGF-β) [18]. However, a different study supports a model in which miR-146a plays a protective anti-inflammatory role in OA [19]. Furthermore, miR-146a was shown to be involved in human chondrocyte apoptosis in response to mechanical injury, and may contribute to the pathogenesis of OA [20]. These findings suggest that miRNAs play critical roles in skeletal development and diseases.

We have identified that mechanical loading up-regulated miR-365 in growth plate chondrocytes. This process promotes chondrocyte differentiation through the inhibition of histone deacetylase 4 (HDAC4) [21], which is a major regulator of cartilage development and endochondral ossification by functioning as a potent inhibitor of chondrocyte hypertrophy. A recent study has shown that decreased HDAC4 is responsible for the increase of Runt-related transcription factor 2 (Runx2) and the OA-related genes in human OA cartilage [22]. In this study, we investigated whether miR-365 is mechanical responsive and how it is regulated in OA cartilage. We aim to address the role of mechanical sensitive miR-365 in OA by using human articular chondrocytes, an animal model and osteoarthritis patients and traumatic osteoarthritis patients. Here, we identify that miR-365 is a mechanically induced mediator of cartilage degeneration. Cyclic loading is sufficient for the transcriptional regulation of miR-365 through NF-κB signaling. The up-regulation of miR-365 further contributes to cartilage catabolic effects in OA through inhibition of its target HDAC4. Understanding the regulation of miR-365 in this pathophysiological condition is of great importance and could open up new therapeutic avenues targeting the disease.

## 2. Results

### 2.1. Cyclic Loading Transcriptionally Up-Regulates miR-365 through NF-кB Signaling

**Figure 1:**
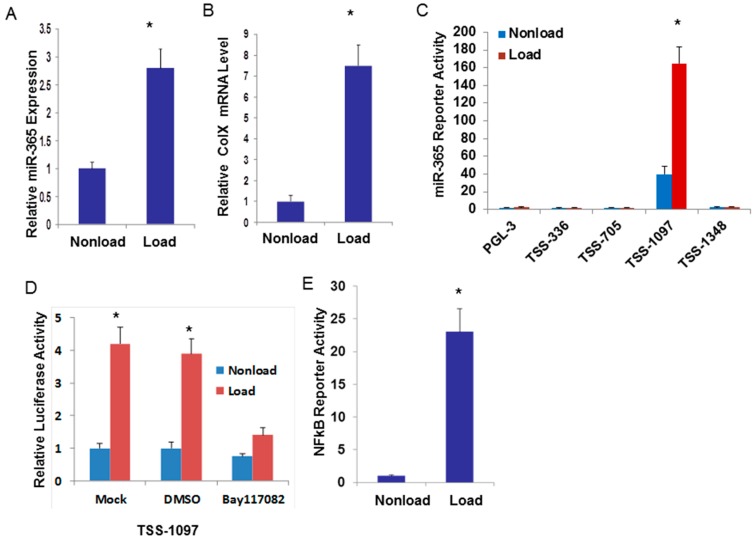
Cyclic loading stimulates transcriptional regulation of miR-365 through NF-κB. (**A**,**B**) Human articular chondrocytes were seeded into 3D collagen sponges and subjected to 10% cyclic loading at 1 Hz for 24 h. Total RNA was extracted and the expression of endogenous miR-365 and Col X was detected by real-time PCR; (**C**) Human articular chondrocytes were cultured in 24-well plates and transfected with miR-365 promoter reporter mutants containing various lengths of the miR-365 promoter region. At 24 h post-transfection, cells were subjected to 10% cyclic loading at 1Hz for 24 h. Luciferase activity of the mock-transfected empty vector pGL3 Basic group was regarded as 1. * *p* < 0.05; (**D**) Human articular chondrocytes were transfected with transcription start site (TSS)-1097-Luc reporter. At 24 h post-transfection, cells were treated with Dimethyl sulfoxide (DMSO) or BAY11-7082, selectively and irreversibly inhibits NF-κB activation for one hour and subjected to 10% cyclic loading at 1 Hz for 24 h. Luciferase activities were measured 24 h later. Luciferase activity of the nonloaded mock-transfected empty vector pGL3 group was regarded as 1. * *p* < 0.05, compared with nonload control; (**E**) Human articular chondrocytes were transfected with NF-κB -Luc reporter. At 24 h post-transfection, cells were subjected to 10% cyclic loading at 1 Hz for 24 h. Luciferase activities were measured 24 h later. Luciferase activity of the nonload control was regarded as 1. * *p* < 0.05.

Human articular chondrocytes isolated from normal looking human OA cartilage were seeded into 3D collagen sponges and subjected to 10% elongation, 1 Hz cyclic loading. The expression of miR-365 was significantly up-regulated by cyclic loading compared with its non-load control (Figure 1A). Mechanical sensitive gene Col X [23] expression is also up-regulated by cyclic loading (Figure 1B).

To further investigate whether mechanical stimulation activates the transcription of miR-365, the upstream of the transcription start site (TSS) of the miR-365 promoter or its mutant containing various lengths of the miR-365 were transfected into chondrocytes and subjected to cyclic loading. Interestingly, luciferase assay results demonstrate that the activity of reporters which is from 1097 to 705 base pairs upstream of the transcription start site (TSS-1097) was significantly induced by cyclic loading (Figure 1C). A previous study showed that two nuclear factor-κB (NF-κB) binding sites were located in this promoter region [24]. We then pretreated with NF-κB specific inhibitor BAY11-7082 for one hour before we applied 10% cyclic loading. Interfering with NF-κB activity by pretreatment with 10 μM BAY11-7082 inhibited transcriptional increase by cyclic loading (Figure 1D). NF-κB signaling is a major catabolic signaling pathway in OA. We then tested the possibility that cyclic loading stimulates NF-κB signaling in human articular chondrocytes. We performed a luciferase reporter assay using the NF-κB-responsive luciferase construct encoding the firefly luciferase reporter gene under the control of a Cytomegalovirus (CMV) promoter and tandem repeats of the NF-κB transcriptional response element. NF-κB luciferase activity was significantly up-regulated in 3D chondrocytes loaded with 10% elongation for 24 h (Figure 1E). Therefore, cyclic loading transcriptionally up-regulates miR-365 through NF-κB signaling.

### 2.2. miR-365 is Up-Regulated by Pro-inflammatory Cytokine IL-1β and is Essential for Cartilage Degeneration

**Figure 2:**
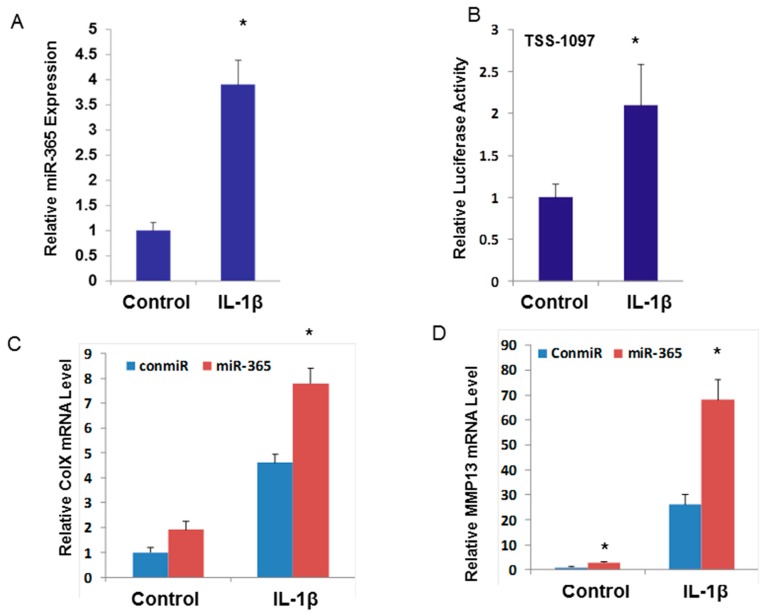
IL-1β stimulates miR-365 and exacerbates the catabolic effect in human articular chondrocytes. (**A**) Articular chondrocytes isolated from human cartilage were treated with 10 ng/mL Il-1β for 24 h. miR-365 expression was determined by real-time PCR. * *p* < 0.05; (**B**) Human articular chondrocytes were transfected with TSS-1097-Luc reporter and treated with 10 ng/mL Il-1β 24 h post transfection. Luciferase activities were measured 24 h later; (**C**,**D**) Human articular chondrocytes were transfected with miR-365 mimic or control mimic and treated with or without Il-1β at 24 h post-transfection. RNA was collected 24 h later and the expression level of collagen type X (Col X) and metallopeptidase 13 (MMP13) was analyzed by real-time PCR. Control mimic transfected without IL-1 treatment was regarded as 1. *n* = 3, * *p* < 0.05.

IL-1β is an important catabolic factor of joint inflammation and cartilage degradation in OA. To investigate whether the expression of miR-365 was induced by IL-1β, primary human chondrocytes were stimulated with IL-1β for 24 h. IL-1β stimulation of chondrocytes resulted in significant up-regulation of miR-365 expression (Figure 2A). Furthermore, luciferase assay on miR-365 promoter activity was increased following treatment with IL-1β (Figure 2B).

Studies have shown that NF-κB signaling may contribute to hypertrophic-like differentiation of chondrocytes in OA. We examined the mRNA level of hypertrophic marker gene Col X. Indeed, overexpression of miR-365 significantly up-regulated Col X and further increase of IL-1β induced the up-regulation of Col X (Figure 2C). A hallmark of OA chondrocytes is their increased production of matrix degrading enzymes. We therefore asked whether miR-365 is sufficient to up-regulate metallopeptidase 13 (MMP13), a crucial effector of OA cartilage destruction. MMP13 was significantly up-regulated by transfection of chondrocytes with miR-365 mimic and was exacerbated by IL-1β treatment (Figure 2D).

### 2.3. miR-365 Expression Is Increased in Osteoarthritis (OA) Cartilage

**Figure 3:**
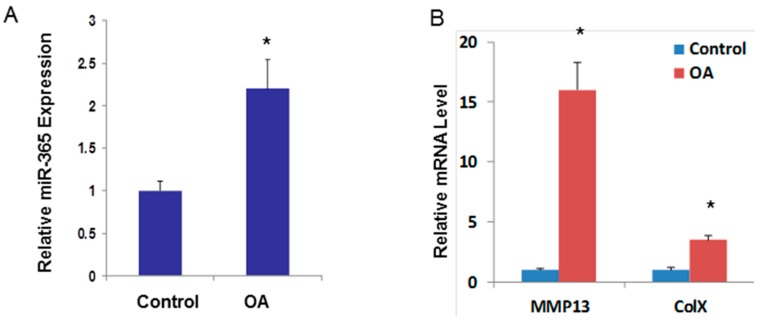

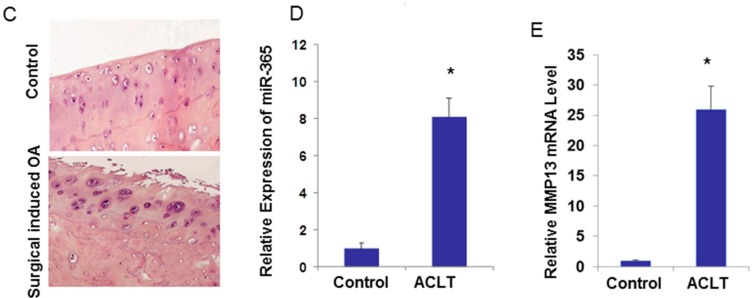
Expression of miR-365 is elevated in articular chondrocytes and experimental osteoarthritis (OA) rats. (**A**) miR-365 expressed at a higher level in OA chondrocytes compared with normal chondrocytes (Control). OA chondrocytes were isolated from cartilage of a 62-year-old OA patient undergoing total knee arthroplasty. Normal control chondrocytes were isolated from 26-year-old male patient undergoing amputation because of trauma; (**B**) The expression level of MMP13 was analyzed by real-time PCR. *n* = 3, * *p* < 0.05; (**C**) Cartilage degradation in anterior cruciate ligament transection (ACLT) surgery induced OA model in rats. Tissue section was stained with H&E staining; (**D**,**E**) miR-365 expression and MMP13 were significantly induced in the cartilage from anterior cruciate ligament (ACL) surgery mice compared with cartilage from sham operated leg (Control). Rat joint cartilage tissues were analyzed by TaqMan miRNA assay for miR-365 expression (**D**) and by SYBR Green real-time polymerase chain reaction for MMP13 mRNA expression (**E**). Results are the mean ± SD, *n* = 6, * *p* < 0.05.

**Figure 4:**
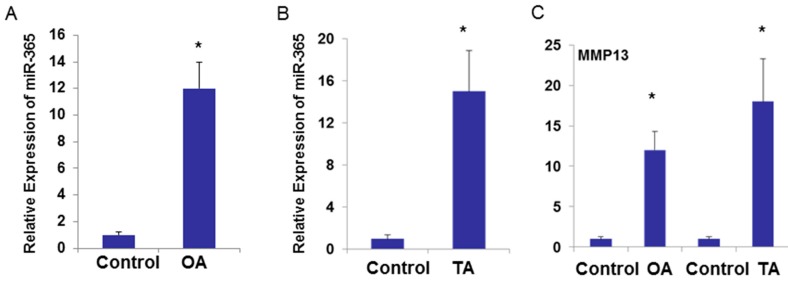
miR-365 is up-regulated in human OA cartilage. (**A**–**C**) RNA was collected with human primary or traumatic OA cartilage in loading area or normal looking cartilage from non-load control cartilage (*n* = 15) and were analyzed by TaqMan miRNA assay for miR-365 expression (**B**,**C**) and by SYBR Green real-time polymerase chain reaction for MMP13 mRNA expression. OA, cartilages are from primary OA patients; TA, cartilage are from traumatic OA. * *p* < 0.05.

To determine whether miR-365 is involved in OA pathogenesis, we compared its expression level between normal and OA chondrocytes. The expression of miR-365 in OA chondrocytes was significantly higher than that in the relative normal chondrocytes (Figure 3A). As expected, MMP13 and Col X were up-regulated in OA chondrocytes (Figure 3B). To further determine whether miR-365 is up-regulated in OA cartilage *in vivo*, we used the surgical arthritis model to experimentally induce OA pathogenesis, in which the articular cartilage was exposed to an excessive mechanical load, due to joint instability caused by surgical resection of the anterior cruciate ligament (ACL) in rats. The joint cartilage in the surgical rats exhibit accelerated proteoglycan loss and fibrillation of articular cartilage in knee joints compared with the control at six weeks post-surgery. Cartilage in the control group does not appear to have pathological changes (Figure 3C). The expression of miR-365 was significantly up-regulated in OA cartilage compared with normal cartilage (Figure 3D). Expression of matrix degrading enzymes MMP13 and ADAM Metallopeptidase with Thrombospondin Type 1 Motif 5 (ADAMTS5) were significantly increased in surgical induced OA cartilage compared with control cartilage as shown by real-time PCR (Figure 3E). We have found a significant difference in miR-365 expression in the OA *vs.* control model in rats. To further confirm relations between OA and miR-365 in human cartilage, we quantify the miR-365 expression level in primary OA (PA) and traumatic OA (TA) groups, which were divided according to the patient’s medical history. We observed the slices harvested from all patients under the microscope and assessed the osteoarthritic damage. To exclude the variation between individuals, we collected OA cartilage in load bearing area and normal looking cartilage from non-loading area in the same patient. The OA cartilage exhibited significant proteoglycan loss compared with the control cartilage (data not shown). The miR-365 expression levels in the lesion area were significantly higher than the corresponding non-lesion control area in both the primary OA and traumatic OA groups (Figure 4A,B). There was significant difference between the TA and PA patients. Expression of MMP13 was significantly increased in OA cartilage compared with control cartilage (Figure 4C).

### 2.4. miR-365 Directly Targets HDAC4 Leading to Down-Regulation of HDAC4 Expression

**Figure 5:**
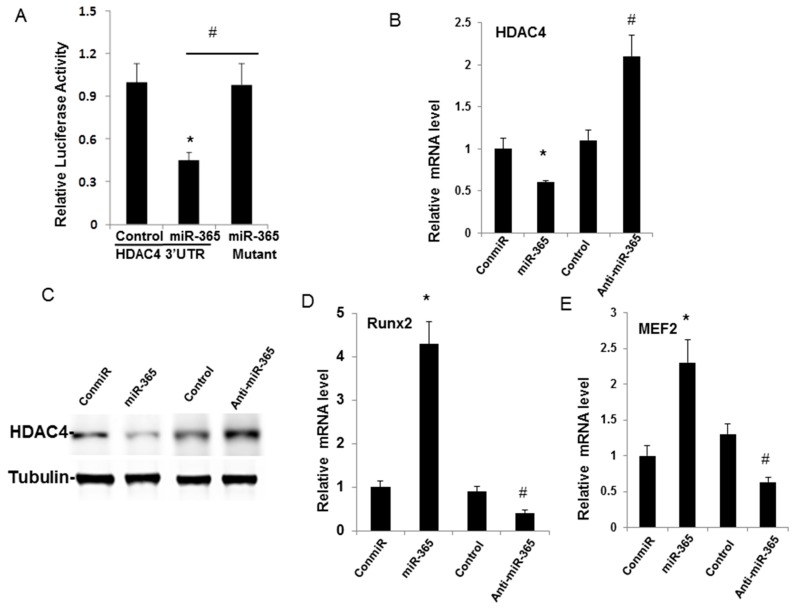
miR-365 directly targets histone deacetylase 4 (HDAC4). (**A**) Overexpression of miR-365 down-regulated luciferase activity of WT HDAC4 3′-UTR, Human articular chondrocytes were co-transfected with a reporter carrying a WT or mutant of HDAC4 3′-UTR along with miR-365 or its control mimics and were analyzed by dual luciferase assay. * *p* < 0.05, compared with the control; # *p* < 0.05, compared with the chondrocytes overexpressed with HDAC4 3′-UTR and miR-365; (**B**,**D**,**E**) Human articular chondrocytes were transfected with miR-365 mimic or miR-365 inhibitor and their control. RNA was collected 48 h post-transfection. mRNA levels of HDAC4, Runx2 or MEF2C were detected with real-time PCR. * *p* < 0.05, compared with control miRNA mimic (ConmiR.); # *p* < 0.05, compared with control miRNA inhibitor (Control); (**C**) Western blot analysis of HDAC4 expression in human articular chondrocytes transfected with miR-365 mimic or miR-365 inhibitor and their control. Tubulin was used as the control.

To further clarify the molecular mechanism underlying miR-365 on OA pathogenesis, we are interested in identifying the direct target of miR-365 that may be involved in OA development. HDAC4 is a direct target in growth plate chondrocytes and a recent study has shown that decreased HDAC4 is responsible for the increase of Runx2 and OA-related genes in human OA cartilage [21,22]. To identify whether HDAC4 is a direct target of miR-365 in human chondrocytes, we transfected a wild-type HDAC4 3′-UTR construct containing a putative miR-365 binding sequence. A Luciferase reporter assay revealed that overexpression of miR-365 significantly inhibited reporter activity in human chondrocytes. Furthermore, we found that the construct bearing mutations at a putative miR-365 binding site completely abolished the inhibitory effect of miR-365 (Figure 5A). Western blotting results further confirmed the inhibition of HDAC4 mRNA and protein expression by miR-365 while anti–miR-365 enhanced the mRNA and protein level of HDAC4 in human chondrocytes (Figure 5B,C). It is known that HDAC4 suppresses chondrocyte hypertrophy by inhibiting Runx2 and MEF2C expression during growth plate development. Consistently, the change of HDAC4 expression regulates its downstream transcription factors such as Runx2 and MEF2C in human chondrocytes (Figure 5D,E).

### 2.5. Inhibition of miR-365 or Overexpression of HDAC4 Impairs IL-1β Induction of MMP13 mRNA

**Figure 6:**
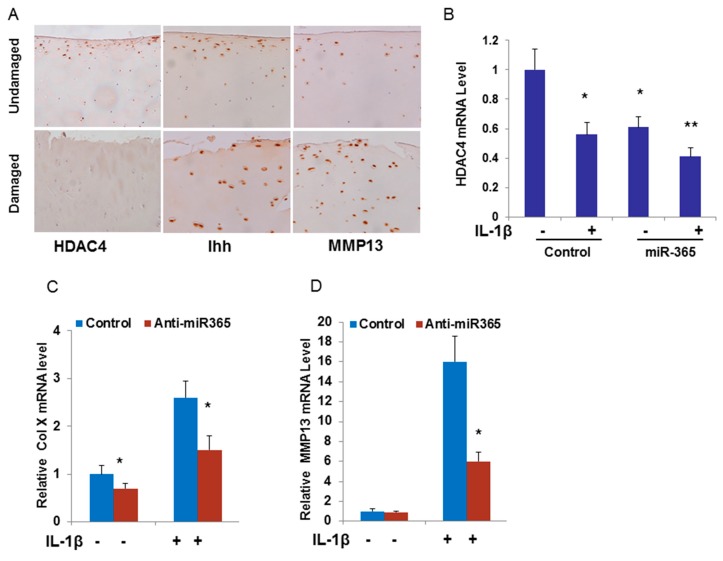
miR-365 targets HDAC4 and mediates the catabolic effect in chondrocytes. (**A**) Immunohistochemical analysis of HDAC4, Indian Hedgehog (Ihh) and MMP13 in cartilage sections from OA patients; (**B**) Human articular chondrocytes were transfected with miR-365 mimic and control mimic for 24 h then treated with (+) or without (−) 10 ng/mL IL-1β for 24 h. RNA was collected and mRNA levels of HDAC4 were detected by real-time PCR. * *p* < 0.05; ** *p* < 0.01, compared with control mimic transfection without IL-1β treatment; (**C**,**D**) Human articular chondrocytes were transfected with anti-miR-365 inhibitor for 24 h then treated with (+) or without (−) 10 ng/mL IL-1β for 24 h. RNA was collected and mRNA levels of Col X and MMP13 were detected by real-time PCR. * *p* < 0.05, compared with control inhibitor transfection.

To further confirm that miR-365 contributes to OA pathogenesis through HDAC4, we examined the expression of HDAC4 in human OA cartilage. We have shown that miR-365 is expressed at a higher level in OA cartilage (Figure 4B,C). Interestingly, HDAC4 is expressed at a lower level in OA cartilage by Immunohistochemistry (IHC) analysis (Figure 6A). This further supports the notion that HDAC4 may be a direct target of miR365, which mediates OA pathogenesis. The expression of MMP13 and Indian Hedgehog (Ihh) were significantly up-regulated in OA cartilage compared with the unloaded control cartilage (Figure 6A). More interestingly, IL-1β significantly reduced expression of HDAC4 mRNA, which resembled the effect of miR-365 overexpressing chondrocytes (Figure 6B). miR-365 overexpression and IL-1β treatment have a synergistic effect on the down-regulation of HDAC4 (Figure 6B). Furthermore, inhibition of miR-365 reduced the IL-1β induced catabolic effect as shown by significant inhibition of MMP13 (Figure 6C). Taken together, these findings suggest that miR-365 directly targets HDAC4 in human chondrocytes and mediates its catabolic effects.

## 3. Discussion

Mechanical stimulation is an important pathogenic factor in OA and is considered to be the major cause of traumatic OA. How mechanical stimulation acts on chondrocytes to mediate biochemical signals, triggering cell differentiation and destruction of cartilage matrix remains poorly understood [1]. Furthermore, OA is no longer thought to be a purely “wear and tear” process but rather one that has been increasingly recognized to include low-grade inflammation. In this study, we explain a possible causal relationship between the mechanical stress and pro-inflammatory stimuli responsive miR-365, which regulates catabolic effects in human articular chondrocytes and mediates OA development. We found that cyclic loading in 3D chondrocytes and disruption of extracellular matrix (ECM) by mechanical instability induced by anterior cruciate ligament transection (ACLT) surgery, triggered increased miR-365 expression in cartilage. Furthermore, miR-365 is transcriptionally up-regulated by cyclic loading and IL-1β stimulation through the activation of NF-κB signaling in the articular chondrocytes. miR-365 is expressed at higher levels in human OA cartilage from primary OA patients as well as OA patients with traumatic history, supporting its pathological significance. Overexpression of miR-365 induces chondrocyte differentiation marker Ihh and MMP13 and exacerbates the IL-1 induced catabolic effects in chondrocytes. We further show HDAC4 is a direct target of miR-365, which mediates mechanical stress and inflammation in OA pathogenesis, thereby leading to accelerated cartilage destruction.

The family of NF-κB transcription factors is intimately involved in the regulation of expression of numerous genes in the setting of the inflammatory response. Many *in vitro* and *in vivo* studies have also indicated the contribution of components of the NF-κB signaling pathways to the pathogenesis of various rheumatic diseases, in particular, osteoarthritis [25,26]. Furthermore, mechanical overload induces similar intracellular events to those generated by pro-inflammatory cytokines in arthritis [27]. This is consistent with our study that cyclic loading and IL-1 both up-regulate the transcription of miR-365 through activation of NF-κB. IL-6 is up-regulated in OA cartilage which is consistent with previous studies that show that NF-κB signal pathways are employed by mechanical signals for transcriptional regulation of IL-6 that are involved in catabolic events in chondrocytes [25,28].

Mechanical force may be the most important single environmental factor responsible for joint homeostasis. These articular cartilage injuries, as a consequence of either acute or chronic high-intensity load will frequently result in cartilage degeneration, which may eventually lead to traumatic OA [29,30]. In this study, we observed mechanical responsive miR-365 is expressed at higher levels in both primary OA and traumatic OA cartilage. However, we failed to conclude that traumatic OA cartilage expresses higher levels of miR-365 compared with primary OA cartilage. This may be due to the fact that clinically recognizable symptoms appear late in the osteoarthritic process. Our understanding of the biology of the disease has been hampered by the lack of access to tissue samples from patients with early stage disease. How the disease is initiated and what factors trigger the disease process remains unclear. However, our data demonstrate miR-365 is induced by mechanical stress as well as an inflammatory cytokine which contributes to the pathological sequence of OA progression.

Histone deacetylases (HDACs) are protein deacetylases with rapidly a growing number of substrates and functions [31,32]. HDAC4-null mice display premature ossification of developing bones due to ectopic and early onset chondrocyte hypertrophy. Wei *et al.* has shown HDAC4 may be involved in the pathogenesis of OA development and demonstrate a statistically negative relationship between HDAC4 expression level and severity of OA [22]. In this study, we showed that HDAC4 is a direct target of miR-365 in human articular chondrocytes at different levels. HDAC4 protein expression is modulated by miR-365, and this regulatory effect is driven by the miR-365 conserved seed sites within the 3′-UTR of HDAC4. As a result, the downstream Runx2 and MEF2C were regulated by miR-365 and contribute to hypertrophic conversion of chondrocytes in OA cartilage. Hypertrophic maturation of chondrocytes is a crucial step in endochondral ossification, whereas abnormally accelerated differentiation of hypertrophic chondrocytes in articular cartilage is linked to pathogenesis of OA. Furthermore, a recent study has shown that HDAC4 is in involved inflammatory cytokine production in macrophages and HDAC4 was degraded after prolonged Lipopolysaccharide (LPS) treatment [33]. Therefore, miR-365 contributes to OA pathogenesis by targeting HDAC4 through two mechanisms: hypertrophic conversion of articular chondrocytes through RUNX2 and MEF2C (Figure 5C) and promoting inflammatory cytokine production to increase catabolic effects.

Although the critical role of miR-365 in cartilage maintenance may be explained largely by identifying HDAC4 as its direct target, miRNAs are believed to regulate multiple target mRNAs. Therefore, the role of miR-365 in cartilage homeostasis may involve the regulation of additional genes. *In vivo* work using transgenic animal models to overexpress miR-365 or knockout miR-365 in cartilage will be needed to establish the definite etiologic answer for miR-365 in OA pathogenesis. Furthermore, there are other mechanisms activated in joint tissues in response to injury and an altered mechanical environment, including altered mechanoreceptor signaling and release of growth factors such as nerve growth factor [34]. It remains unknown whether this involves regulation of miR-365 or other mechanical sensitive miRNAs.

In conclusion, our results collectively indicate that miR-365 acts as a mechanically induced mediator of osteoarthritic cartilage destruction, by directly regulating HDAC4. Mechanical overload is an important risk factor in OA pathogenesis. Furthermore, in the late stage of OA, stress from the combination of overload and inflammatory cytokines further increases miR-365, leading to cartilage degradation. Our finding facilitates the understanding of the pathogenesis of degenerative cartilage and provides a potential therapeutic strategy for the regulation of osteoarthritic cartilage destruction. Manipulation of the expression of miR-365 in articular chondrocytes by a miR-365 inhibitor may be a potent therapeutic for the prevention and treatment of OA.

## 4. Materials and Methods

### 4.1. Primary OA and Traumatic OA Specimen Selection

Human articular cartilage was aseptically obtained at the time of total knee replacement from OA patients. All individuals satisfied the American College of Rheumatology Classification Criteria for OA [35]. This study was performed with ethics committee approval (qddx-2011-21), and all patients provided informed consent. The OA patients were separated by primary arthritis group and traumatic arthritis group based upon clinical history and examination coupled with radiographic findings. Primary osteoarthritis group: Patient ages range from 59 to 67 years (62.7 ± 2.4, *n* = 15; 13 women and 2 men). Traumatic arthritis group: Patient ages range from 53 to 60 years (56.2 ± 2.3, *n* = 12; 6 women and 6 men). These patients had a clear history of trauma; clinical features support the diagnosis of traumatic arthritis, excluding rheumatoid arthritis and other precipitating factors. Osteoarthritic cartilage of the experimental group was harvested around the medial femoral condylar area, which shows gross lesions. The control cartilage was harvested from normal looking non-loaded area in medial femoral epicondyle from the same patient. The cartilage was examined macroscopically and microscopically to ensure that relative normal tissue was used as control. All of the cartilage were full-layer harvested and were fixed in formalin for H&E staining. The cartilage slices were observed under the microscope and the osteoarthritic damage score was assessed individually by three people. Samples were fixed with 4% paraformaldehyde or were preserved in sample stabilizer, RNA later (Thermofisher Scientific, Waltham, MA, USA) and stored at −80 °C for later RNA extraction.

### 4.2. Primary Chondrocyte Culture, Transfection, IL-1β Treatment and 3D Cyclic Loading

Primary human chondrocytes were isolated from articular cartilage samples that were obtained from primary OA patients at the time of total joint arthroplasty (*n* = 6, 5 female and 1 male, age 58–62). Cartilage samples were individually handled and processed separately to isolate chondrocytes. Each cartilage sample was washed, sliced and minced into small fragments then were digested in 2.0 mg/mL of Pronase (Roche, Indianapolis, IN, USA) in Hank’s Balanced Salt Solution (HBSS) for 30 min at 37 °C under shaking conditions. The digestion solution was removed and cartilage was washed twice with DMEM/F-12 medium (Life Technologies, Grand Island, NY, USA) and subsequently digested with 1 mg/mL of Type IA Crude Bacterial Collagenase (Sigma-Aldrich, St. Louis, MO, USA) for 8 h at 37 °C under shaking conditions. The collagenase enzyme reaction was stopped by adding DMEM/F-12 medium containing 10% fetal bovine serum (FBS) (Life Technologies). Solution was filtered using a 100 μm nylon cell strainer (BD, Franklin Lakes, NJ, USA) to remove clumps, followed by centrifugation at 1500 rpm to pellet the successfully isolated chondrocytes. Pellet was washed twice with DMEM/F-12 plus 10% FBS medium and cells were counted using a hemocytometer. Human articular chondrocytes were seeded at high density (10^5^/cm^2^) and cultured in DMEM/F-12 containing 10% FBS and 1% penicillin/streptomycin (Life Technologies) at 37 °C in a humidified atmosphere of 5% CO_2_. Primary chondrocytes were used when comparing expression levels in normal and OA chondrocytes and the first passage chondrocytes were used for all other experiments involving cultured chondrocytes. The medium was changed every three days. When the primary chondrocytes reached 90% confluence, the cells were seeded into 3D collagen sponges as describe previously [21] or 6-well plates for IL-1β treatment or transfection. The cyclic mechanical loading was induced at 10% elongation at 60 cycles/min, 15 min/h by a computer-controlled biostretch device (Bio-Stretch; ICCT Technologies, Markham, ON, Canada) as described previously [21]. Primary cultured human chondrocytes were serum starved overnight and then treated with 10 ng/mL recombinant human IL-1β (Roche, Branchburg, NJ, USA) for 24 h. Human miR-365 mimic, miR-365 inhibitor and their negative control mimics or inhibitors were obtained from Dharmacon (Thermofisher Scientific). Human articular chondrocytes were transfected with each oligonucleotides using Lipofectamine 2000 (Life Technologies) according to the manufacture’s instructions at a final concentration of 80 nM. Forty-eight hours after transfection, cells were harvested and subjected to total RNA and protein extraction.

### 4.3. Luciferase Assays

The luciferase reporter plasmid TSS-1348, TSS-1097, TSS-705, and TSS-336, which contains the 1348-, 1097-, 705-, and 336-bp proximal promoter sequences upstream of TSS of miR-365, respectively, were a generous gift from Professor Liu-Rong Fang from Hua-Zhong agriculture University, Wuhan, China [24]. pGL-HDAC4 3′-UTR-luciferase reporter generous gift from Dazhi Wang from Harvard University, Boston, USA [36]. For construction of the 3′-UTR, the multiple cloning site of the pGL3-Control vector (Promega, Madison, WI, USA) was removed and placed downstream of the luciferase gene. The 3′-UTRs of HDAC4 was amplified by PCR and cloned them into the modified pGL3-Control vector, resulting in the constructs HDAC4-3′-UTR. pGL-HDAC4-mut was derived from pGL-HDAC4 by mutating 3 bases pairs within miR-365 seed sites within the HDAC4 3′-UTR sequence using the QuikChange XL Site-directed Mutagenesis kit (Stratagene, La Jolla, CA, USA). Mutations were generated by replacing 3 consecutive base pairs at the 3′-UTR region of the seed site as described in our previous paper [21]. The NF-κB Reporter kit was purchased from Qiagen (Qiagen, Valencia, CA, USA). NF-κB-responsive luciferase construct encodes the firefly luciferase reporter gene under the control of a minimal (m) CMV promoter and tandem repeats of the NF-κB transcriptional response element (TRE). For luciferase assay, cells were cultured in 12-well plates and transfected with 0.2 µg of an indicated reporter plasmid or NF-κB reporter construct as well as the negative controls per well and 0.05 µg pRL-TK (Promega) alone with miRNA mimics (50 nM). Transfection was performed using Lipofectamine 2000 (Life Technologies) per manufacturer’s instructions. Luciferase activities were determined using the Dual-Luciferase reporter assay system (Promega) using the dual luciferase assay reporter-ready luminometer. Renilla luciferase activity in the lysates was used to normalize the firefly luciferase activity. The assays were performed in triplicate.

### 4.4. Quantification of mRNA and miRNA

Total RNA in chondrocytes was extracted and quantified as described [21]. Total RNA of cartilage tissue was isolated from fresh-frozen cartilage by grinding the tissue under liquid nitrogen in a freezer mill and extracting the homogenate using the mirVana miRNA isolation kit (Life Technologies). The concentration and the quality of total mircoRNA were determined by Nanodrop. miR-365 expression level was quantified with the Taqman microRNA assays specific for mature miR-365 (Life Technologies). Briefly, 10 ng RNA was transcribed using Taqman microRNA RT Kit (Life Technologies) and followed by real-time PCR with Taqman MicroRNA Assays. The ubiquitously expressed miRNA, snoRNA U6 was used as an endogenous control. The mRNA levels were quantified by real-time PCR with the SYBR Green PCR Master mix (Qiagen). The sequences of the human specific primers used are listed as following: HDAC4 primers: forward, 5′-GAGAGACTCACCCTTCCCG-3′, and reverse, 5′-CCGGTCTGCACCAACCAAG-3′. Runx2 primers: forward, 5′-GGCAGGCACAGTCTTCCC-3′, and reverse, 5′-GGCCCAGTTCTGAAGCACC-3′. MEF2C primers: forward, 5′-TTCCAGTATGCCAGCACCG-3′, and reverse, 5′-GGCCCTTCTTTCTCAACGTCTC-3′. Type X collagen primers were as follows: forward, 5′-GCACGCAGAATCCATCTGAGAATA-3′, and reverse, 5′-GACCAGGAGTACCTTGCTCTC-3′. MMP-13 primers were as follows: forward, 5′-GAAATGCAGTCTTTCTTCGG-3′, and reverse, 5′-GCCTTTTCGACTTCAGAATG-3′. Rat MMP13 primers: forward, 5′-CACCATGCATTCAGCTATTC-3′, and reverse, 5′-CAAGAGTCACAGGATGGTAG-3′. Amplification conditions were as follows: 10 min at 95 °C for enzyme activation; 40 cycles at 95 °C denaturation for 10 s; 55 °C annealing for 30 s; and 72 °C extension for 30 s. 18S ribosomal RNA was used as an internal control gene to normalize the mRNA levels. The cycle threshold (*C*_t_) values for 18s RNA and that of target genes were measured and calculated by computer software (IQ50, Bio-Rad Laboratories, Hercules, CA, USA). Relative transcription levels were calculated as *x*  =  2^−ΔΔ*C*t^, was used for the calculation of fold amplification.

### 4.5. Surgically Induced OA Model by Anterior Cruciate Ligament Transection (ACLT) in Rats

Animal handling and experimental procedures were performed following approval from the Institute of Health Sciences Animal Care and Use Committee. Surgical OA animal model was induced as previously described [37]. Briefly, 12-week-old male healthy Wistar rats were randomly divided into two groups. The anterior cruciate ligament (ACL) was transected to induce abnormal mechanical loading-associated osteoarthritis of the left knee. Sham operation was done by opening the joint capsule and then suturing the incision in the left knee of independent rats. Animals were sacrificed at 6 weeks post-injection or post-surgery, and samples of the knee joints were collected for further molecular and histological analyses. We performed histological analysis using H&E staining and graded articular cartilage degeneration using the Osteoarthritis Research Society International (OARSI)-modified Mankin criteria [38,39]. The osteoarthritic damage score was assessed individually by 3 observers using light microscopy.

### 4.6. Histology and Immunohistochemistry

Knee joints from the rats and human knee cartilage tissues were fixed overnight with 4% paraformaldehyde, decalcified, dehydrated, and then embedded in paraffin. Serial midsagittal tissue sections (5 μm) were cut and deparaffinized in xylene, serially rehydrated in ethanol, and washed with PBS. Sections were then stained with Safranin O/fast green or Hematoxilin and Eosin to identify cartilage degeneration [19]. The scoring of osteoarthritic damage in rats was evaluated by the following criteria: 0, Intact cartilage; 1, Superficial fibrillation; 2, Defects extending to uncalcified cartilage; 3, Defects extending to calcified cartilage; 4, Defects extending to subchondral bone. Immunohistochemistry was carried out using the Histostain plus 3rd Gen IHC Detection kit (Life Technologies). The sections of knee joints were de-paraffinized and re-hydrated through conventional methods. Endogenous peroxidase was blocked by treating the sections with 3% hydrogen peroxide (Sigma-Aldrich) in methanol for 30 min. The sections were digested by 100 mg/mL hyaluronidase (Sigma-Aldrich) for 15 min. Nonspecific protein binding was blocked by incubation with a serum blocking solution. The sections were then immunostained using antibodies against ADAMTS5 (Abcam, Cambridge, MA, USA), Ihh (Abcam), MMP13 (Abcam), HDAC4 (Santa Cruz biotechnology Shanghai, Shanghai, China) and IL-6 (Santa Cruz biotechnology Shanghai) at 4 °C overnight. The negative control sections were incubated with isotype control (Santa Cruz biotechnology, Dallas, TX, USA) in 0.01 M PBS. Thereafter, the sections were treated sequentially with ready to use biotinylated secondary antibody and ready to use streptavidin-peroxidase conjugate, followed by standardized development in 3,3′-Diaminobenzidine (DAB) chromogen. The sections were counterstained with ready to use hematoxylin (Life Technologies). Photography was performed with a Nikon E800 microscope (Melville, NY, USA).

### 4.7. Western Blot Analysis

Total protein was extracted from human articular chondrocytes 48 h post-transfection of miR-365 mimic or inhibitor and their controls. Equal amounts of cell lysates were loaded in SDS-polyacrylamide gel electrophoresis (SDS-PAGE) and transferred to nitrocellulose membrane, as described previously [21]. Immunoblotting was coupled with fluorescent signal detection using an Odyssey fluorescence scanner (LI-COR Biosciences, Lincoln, NE, USA). The following antibodies were used for this study: anti-HDAC4 antibody and anti-tubulin antibody purchased from Santa Cruz biotechnology, anti-mouse-IRDye800 (Roche; Rockland Immunochemicals, Gilbertsville, PA, USA) and anti-rabbit-Alexa Fluor 680 (Molecular Probes, Eugene, OR, USA).

### 4.8. Statistical Analysis

Results obtained from three independent experiments were expressed as mean ± S.D (standard deviation). Statistical analysis was carried out using two-tailed Student’s *t* test between two groups or one-way analysis of variance followed by Student-Newman–Keuls test for multiple comparisons. *p* < 0.05 was considered statistically significant.
